# Survey on Knowledge, Attitudes, and Training Needs of Italian Residents on Genetic Tests for Hereditary Breast and Colorectal Cancer

**DOI:** 10.1155/2014/418416

**Published:** 2014-06-23

**Authors:** Nikola Panic, Emanuele Leoncini, Paolo Di Giannantonio, Benedetto Simone, Andrea Silenzi, Anna Maria Ferriero, Roberto Falvo, Giulia Silvestrini, Chiara Cadeddu, Carolina Marzuillo, Corrado De Vito, Walter Ricciardi, Paolo Villari, Stefania Boccia

**Affiliations:** ^1^Section of Hygiene, Department of Public Health, Institute of Public Health, Università Cattolica del Sacro Cuore, Largo F. Vito 1, 00168 Rome, Italy; ^2^University Clinical-Hospital Center “Dr. Dragisa Misovic-Dedinje”, Milana Tepica 1, 11000 Belgrade, Serbia; ^3^Department of Public Health and Infectious Diseases, Sapienza University of Rome, Piazzale Aldo Moro 5, 00185 Rome, Italy; ^4^IRCCS San Raffaele Pisana, Via della Pisana 235, 00163 Rome, Italy

## Abstract

*Objectives.* The aim of the study was to assess knowledge and attitudes of medical residents working in Università Cattolica del Sacro Cuore, Rome, Italy, on genetic tests for breast and colorectal cancer. *Methods.* We distributed self-administered questionnaire to the residents. Logistic regression models were used to evaluate the determinants of knowledge and attitudes towards the tests. *Results.* Of 754 residents, 364 filled in questionnaire. Around 70% and 20% answered correctly >80% of questions on breast and colorectal cancer tests, respectively. Knowledge on tests for breast cancer was higher among residents who attended course on cancer genetic testing during graduate training (odds ratio (OR): 1.72; 95% confidence interval (CI): 1.05–2.82) and inversely associated with male gender (OR: 0.55; 95% CI: 0.35–0.87). As for colorectal cancer, residents were more knowledgeable if they attended courses on cancer genetic testing (OR: 2.08; 95% CI: 1.07–4.03) or postgraduate training courses in epidemiology and evidence-based medicine (OR: 1.95; 95% CI: 1.03–3.69). More than 70% asked for the additional training on the genetic tests for cancer during the specialization school. *Conclusion.* The knowledge of Italian residents on genetic tests for colorectal cancer appears to be insufficient. There is a need for additional training in this field.

## 1. Introduction

Since the first global look at the content of the human genetic code, published more than a decade ago [1], it became clear that the implementation of genomic medicine has the potential of bringing clinically important advances, to rival those of any other major discovery in the history of medicine [2]. In this context, doctors have been envisaged as the key players in properly incorporating emerging DNA technologies in the health care system [3, 4], leading to calls for enhanced genomic education for healthcare professionals. Nevertheless, several studies conducted in USA, Europe, and Canada showed unsatisfactory level of clinicians' ability to use genetic tests in clinical care [3–5].

Recent research showed that providing genetic educational outreach to doctors has a positive impact in improving their competency and confidence in the use of genetic testing to guide prevention or treatment decisions [6–8]. Although the importance of genomic education for health care professionals has been recognized even before completion of the Human Genome Project [9], many physicians do not feel to have a proper training and knowledge [3, 4].

Genetic tests for breast and colorectal cancer, if used appropriately, have been demonstrated to be efficacious and cost-effective [10]. However, Marzuillo et al. reported a significant lack of knowledge on* BRCA1/2* and* APC* tests among Italian medical doctors [5], irrespective of their specialty. Along similar lines, we conducted survey on Italian doctors attending postgraduate medical schools in order to examine the level of education they had received on genetic tests for breast and colorectal cancer during their recent medical training.

More specifically, our objective was to assess knowledge, attitudes, and educational needs of Italian residents related to the use of genetic tests for breast and colorectal cancer, particularly the* BRCA1/2* and* APC* tests, irrespective of the field of specialization.

## 2. Methods

A self-administered anonymous questionnaire was distributed in 2011 to all the residents enrolled at the Gemelli Teaching Hospital of the Università Cattolica del Sacro Cuore in Rome, Italy. The total number of eligible participants was 754, and the number of surveyed postgraduate schools was 43.

A similar questionnaire was previously used and validated in a study conducted on a sample of Italian medical specialists [5]. It comprises questions designed to assess (i) demographic and professional characteristics; (ii) knowledge and attitudes towards genetic tests for hereditary breast and colorectal cancer; (iii) self-assessed level of knowledge and training needs.

Knowledge of genetic tests for breast and colorectal cancer was investigated with three questions, each using a three-point options Likert scale (“agree,” “uncertain,” and “disagree”). Additional four multiple-choice questions were designed to evaluate the residents' knowledge of prevalence of hereditary breast cancer and inherited forms of colorectal cancer and of penetrance of* BRCA1/BRCA2 *and* APC* mutations (two each).

Attitudes towards genetic tests for breast and colorectal cancers were assessed with seven different questions, also using a Likert scale evaluation.

The final set of questions required the residents to assess their own perceived level of knowledge according to a four-answer format (“inadequate,” “sufficient,” “good,” and “excellent”), and training needs (“yes/no” answer).

### 2.1. Statistical Analysis

A descriptive analysis was conducted to report demographic, social, and professional characteristics of responding residents. For the questions on knowledge and attitudes of residents towards genetic testing for breast and colorectal cancer, we calculated the proportions of correct answers (plus 95% confidence intervals (CI)). We considered residents who gave 80% correct answers for each form of hereditary cancer as knowledgeable. A general positive attitude towards predicative genetic testing was defined as the presence of a positive attitude in at least 70% of the questions assessing the attitude. Variables associated (*P* value of <0.20) with a positive outcome (satisfactory knowledge/attitude) from the univariate analysis were included in the multivariate conditional logistic regression model. A backward elimination procedure was used for the multivariate analysis. Data were analyzed using Stata software (StataCorp. 2009. Stata Statistical Software: Release 11. College Station, TX: StataCorp LP).

## 3. Results

Of the initial number of 754 eligible for inclusion, 364 residents responded (overall response rate 48.3%).

The demographic and professional characteristics of responding residents are reported in Table 1. Among respondents, 61.2% (222/364) were female; the age mode was of 28-29 years (151/364, 41.6%) and 63.1% (224/364) have had their specialization associated with clinical activity.

Majority of included residents (67.3%, 245/364) answered correctly at least 80% of questions on genetic tests for breast cancer, prevalence of hereditary forms, and penetrance of* BRCA* mutations. Residents knowledgeable on genetic tests for colorectal cancer, prevalence of hereditary forms, and penetrance of* APC* mutations were 21.2% (77/364).  Table 2 reports the correct answer rates in relation to each particular question. The knowledge on test for breast cancer was higher among residents who reported having attended a specific cancer course on genetic testing during their graduate training (OR: 1.72; 95% CI: 1.05–2.82) (Table 3). Male gender was inversely associated with knowledge on tests for breast cancer (OR: 0.55; 95% CI: 0.35–0.87) (Table 3). Residents who attended a specific cancer course on genetic testing during the graduate training (OR: 2.08; 95% CI: 1.07–4.03) as those who took postgraduate training courses in epidemiology and evidence-based medicine (EBM) (OR: 1.95; 95% CI: 1.03–3.69) showed higher knowledge on test for colorectal cancer (Table 3).

Residents' attitudes towards genetic testing for breast and colorectal cancer are reported in Table 4. A total of 45.6% showed positive attitude in at least 70% of questions. Less than half appeared to comply with principles of efficacy (attitude number 2, 48.3%) and cost-effectiveness (attitude number 3, 46.3%) in this field (Table 4). When we analyzed predictors of positive attitude towards genetic testing; only personal family history of breast and/or colorectal cancer (OR: 1.74; 95% CI: 1.11–2.71) appeared to be significantly associated.

Table 5 reports the self-estimated level of knowledge on genetic tests for breast and colorectal cancer and training needs of participants. More than half of our sample (178/364, 50.6%) described their knowledge as poor and 84.6% (296/364) declared that they did not feel qualified to prescribe genetic tests (Table 5). Conversely, 87.8% (309/364) of them declared that is was important for them to increase their knowledge in this field (Table 5). The majority of residents felt that more training time should be allotted to genetic testing during medical studies (289/364, 82.3%) or specialization school (263/364, 74.7%) or through specific postgraduate courses (289/229, 65.4%).

## 4. Discussion

Our study reports unsatisfactory level of residents' knowledge on genetic tests for colorectal cancer and on prevalence of hereditary forms and penetrance of* APC* mutations. We identified female gender and attendance to cancer genetic testing courses during graduate training to be predictors of a better knowledge of genetic tests for breast cancer. An attendance to cancer genetic testing courses during the graduate training and postgraduate training courses in epidemiology end EBM were associated with better knowledge on genetic tests on colorectal cancer. Although the vast majority of participants recognized the important role of genetic tests in prevention, as well as the need for evidence-based guidelines, complex prevention strategies, and genetic counseling, the principles of efficacy and cost-effectiveness appear to be not so widely accepted. The self-assessment revealed that participants are not satisfied with their own knowledge of genetic test and that they do not feel qualified to prescribe them. However, the need for training in this field during graduate and postgraduate studies was clearly recognized.

Some surveys already reported lack of knowledge among medical doctors on genetic tests for cancer [5, 11–20]. Nevertheless, most of these were conducted among specialists, while only one referred to residents [18]. Younger age [20] and recent graduation from medical school [21], as well as being in medical practice less than 10 years [11], have been previously reported as predictors of better knowledge on genetic tests and increased confidence in using them in everyday practice. Marzuillo et al. [5] previously reported insufficient knowledge on genetic test on breast and colorectal cancer among Italian specialists. Although our study showed relatively more satisfactory results in relation to tests for breast cancer, knowledge on genetic tests on colorectal cancer among residents in our study was also fairly low, indicating the need for further improvement in specialists formatting process. Finally, our results clearly pointed the need for additional education in field of genomics as exposure to genetic test during graduate training as well as postgraduate training courses in epidemiology and EBM were associated with higher knowledge on genetic test for breast and colorectal cancer.

Attitude of medical doctors is crucial for the dissemination and implementation of new medical technologies. Although residents in our study have shown high rates of some individual positive attitudes towards genetic testing, only a minority showed positive attitude in all issues. Furthermore, the majority of residents do not recognize the importance of the principles of efficacy and cost-effectiveness in genetic testing. Similar results were obtained from the survey on Italian specialists, who also did not show cost-conscious behavior regarding genetic tests [5]. This could lead to introducing of genetic test in clinical practice for commercial purposes only. Having that in mind, specific educational programs and trainings are needed in order to promote more cost-conscious behavior of physicians.

We found that family history of breast and/or colorectal cancer was a significant predictor of positive attitude towards genetic testing. This is to be expected, as personal experiences represent a major influence in determining individual attitudes, and those with positive family cancer history were personally motivated to find out more on genetic tests.

Residents in our study have deemed their knowledge of genetic tests for breast and colorectal cancer insufficient. Insufficient level of knowledge on genetic test has been previously self-reported among medical doctors several times [5, 15]. Our residents also reported a high level of interest in additional training in this field. As earlier studies also reported readiness of physicians to attend additional courses on genetic testing [5, 11, 15, 18], an organized approach to genomics education is needed in order to make the best use of available genetic testing resources.

Our study has some limitations. Firstly, we have conducted our research among residents working in the same hospital, so the results may not be reflecting the knowledge and attitudes of the Italian residents' population. Secondly, our nonresponse rate was relatively high; thus we do not have data on age and gender structure of nonresponders. Although it is not likely that age could differ between responders and nonresponders, as most of the residents belong to the same age group, the difference in gender structure may be an issue as we recognized gender to be factor for knowledge in some specific fields. Nevertheless, our study is, to our knowledge, first in Europe reflecting the knowledge and attitudes of residents on genetic tests and can be valuable in assessing knowledge, attitudes, and educational needs of young doctors in training on a wider scale.

In conclusion, knowledge of Italian residents on genetic tests for colorectal cancer appears to be insufficient. There is a need for additional training in field of genetic tests during graduate and postgraduate studies as well as during specializations. The principles of efficacy and cost-effectiveness in genetic testing are not fully accepted among residents. Specific educational programs are needed in order to promote more cost-conscious behavior.

## Supplementary Material

Supplementary Table S1 contains a list of medical specializations in Italy according to each particular area. The specializations are placed in three different groups referring to “specializations in area of medicine”, “specializations in area of medicine” and “other specializations”.

## Figures and Tables

**Table 1:** The demographic and professional characteristics of responding residents (*n* = 364).

Variables	n	%
Gender		
Male	141	38.8%
Female	222	61.2%
Age (years)		
<28	78	21.5%
28-29	151	41.6%
30-31	84	23.1%
≥32	50	13.8%
Professional area^i^		
Medicine	147	40.4%
Surgery	47	12.9%
Others	170	46.7%
Clinical activity		
No	131	36.9%
Yes	224	63.1%
Exposure to cancer genetic testing during graduate training		
No	95	26.7%
Yes	261	73.3%
Postgraduate training courses in epidemiology and EBM		
No	292	83.0%
Yes	60	17.1%
English language knowledge		
Very low	9	2.5%
Low	38	10.6%
Intermediate	130	36.3%
Good	153	42.7%
Excellent	28	7.8%
Hours per week dedicated to continuing medical education		
<1	48	13.5%
1–5	212	59.4%
6–10	73	20.5%
>10	24	6.7%
Patient request of cancer genetic tests in the previous year∗		
No	162	74.0%
Yes	57	26.0%
Personal or family history of breast cancer		
No	292	81.8%
Yes	65	18.2%
Personal or family history of colorectal cancer		
No	285	80.1%
Yes	71	19.9%
Promotional material about breast cancer received in the previous year		
No	311	86.9%
Yes	47	13.1%
Promotional material about colorectal cancer received in the previous year		
No	325	91.0%
Yes	32	9.0%

EBM: evidence based medicine.

^i^List of specializations according to each area is available in Supplementary Material S1 available online at http://dx.doi.org/10.1155/2014/418416.

∗The number of responders was 219 as only physicians with clinical activity were included.

**Table 2:** Knowledge of residents (*n* = 364) on genetic tests for breast and colorectal cancer, prevalence of hereditary forms, and penetrance of *BRCA1/2 *and *APC* mutations.

	Number of responders to the question	% of correct answers	CI 95%
*BRCA1/2*			
Genetic tests for BRCA1/BRCA2 mutations are able to identify patients at high risk to develop breast cancer (**agree**, uncertain, disagree)	357	93.3	90.2–95.6
The percentage of breast cancer cases associated with mutations in BRCA1/BRCA2 is **1**–**10%**, 15–35%, >35%	354	42.9	37.7–48.3
The absolute risk of developing breast cancer in presence of BRCA1/BRCA2 mutations is <10%, **40**–**80%**, 100%	356	80.3	75.8–84.3
Women with breast cancer and strong family history should perform BRCA1/BRCA2 testing (**agree**, uncertain, disagree)	356	78.7	74.0–82.8
Scientific evidence recommend for BRCA1/BRCA2 positive women clinical and instrumental surveillance starting from the age of 25 (**agree**, uncertain, disagree)	358	84.4	80.2–88.0
*APC*			
Genetic tests for APC mutations are able to identify patients who will develop colorectal carcinoma (**agree**, uncertain, disagree)	355	77.7	73.1–82.0
The percentage of colon cancer cases associated with APC mutations is **<5%**, 10–25%, >40%	352	31.8	27.0–37.0
The absolute risk of developing colorectal cancer in presence of APC mutations is <10%, 40–80%, **100%**	351	27.9	23.3–32.9
APC testing is recommended for 10–12 years old children with a first degree relative with known APC mutation (**agree**, uncertain, disagree)	357	57.4	52.1–62.6
Scientific evidence recommend for APC positive individuals periodic colonoscopy starting from the age of 10–15 (**agree**, uncertain, disagree)	356	55.9	50.6–61.1

Correct answers are in bold.

**Table 3:** Sociodemographic and professional characteristics associated with knowledge on genetic testing for breast cancer (*BRCA1/BRCA2* mutations) and colorectal cancer (*APC* mutations).

	Breast cancer				Colorectal cancer			
	OR	95% CI	OR adj∗	95% CI	OR	95% CI	OR adj^*γ*^	95% CI
Gender								
Female	1.00		1.00		1.00		1.00	
Male	0.53	0.34–0.83	0.55	0.35–0.87	1.24	0.74–2.06	1.36	0.80–2.31
Age								
<28	1.00		1.00		1.00		1.00	
28-29	0.72	0.39–1.32	0.66	0.35–1.24	0.78	0.42–1.43	0.74	0.39–1.41
30-31	0.70	0.36–1.37	0.62	0.31–1.26	0.32	0.14–0.73	0.25	0.10–0.61
≥32	0.71	0.33–1.54	0.68	0.30–1.56	0.39	0.15–0.99	0.33	0.12–0.92
Personal or family history of breast or colon cancer								
No	1.00		1.00		1.00		1.00	
Yes	1.40	0.77–2.57	1.21	0.65–2.25	0.98	0.52–1.86	0.91	0.47–1.77
Professional area^~^								
Medicine	1.00		1.00		1.00		1.00	
Surgery	0.60	0.31–1.17	0.80	0.39–1.64	1.10	0.50–2.40	1.24	0.55–2.80
Others	1.16	0.72–1.87	1.20	0.74–1.96	0.90	0.52–1.55	0.88	0.50–1.55
Clinical activity								
No	1.00		1.00		1.00		1.00	
Yes	0.94	0.59–1.50	0.97	0.60–1.57	0.93	0.55–1.58	1.02	0.59–1.76
Exposure to cancer genetic testing during graduate training								
No	1.00		1.00		1.00		1.00	
Yes	1.73	1.06–2.82	1.72	1.05–2.82	2.01	1.05–3.84	2.08	1.07–4.03
Postgraduate training courses in epidemiology and EBM								
No	1.00		1.00		1.00		1.00	
Yes	0.90	0.50–1.61	0.88	048–1.60	1.85	1.01–3.45	1.95	1.03–3.69
Patient request of cancer genetic tests in the previous year^i^								
No	1.00		1.00		1.00		1.00	
Yes	2.15	1.05–4.38	1.84	0.89–3.83	0.90	0.42–1.92	0.84	0.38–1.84
Hours per week dedicated to continuing medical education								
<1	1.00		1.00		1.00		1.00	
1–5	1.45	0.76–2.75	1.39	0.71–2.73	1.31	0.57–3.00	0.98	0.41–2.30
6–10	2.03	0.93–4.41	2.11	0.93–4.77	1.64	0.65–4.14	1.25	0.48–3.29
>10	1.73	0.61–4.96	1.84	0.62–5.43	1.32	0.38–4.56	0.74	0.19–2.91
Promotional material about breast or colon cancer received in the previous year								
No	1.00		1.00		1.00		1.00	
Yes	1.29	0.65–2.56	1.12	0.56–2.25	0.67	0.25–1.82	0.41	0.14–1.25

OR: odds ratio; CI: confidence interval; EBM: evidence based medicine.

∗OR adjusted by professional area, exposure to cancer genetic testing during graduate training.

^*γ*^OR adjusted by gender, postgraduate training courses about epidemiology and EBM.

^~^List of specializations according to each area is available in Supplementary file S1.

^i^Included physicians with clinical activity.

**Table 4:** Attitudes of residents (*n* = 364) towards genetic testing for breast and colorectal cancer.

	Number of responders to the question	% of correct answers	CI 95%
(1) Genetic tests for breast cancer and colorectal cancer increase the chances of prevention opportunities (**agree**, uncertain, disagree)	355	85.1	80.9–88.6
(2) Genetic tests that able to identify an increased risk of developing breast or colorectal cancer should be performed even if there are no preventive and/or curative interventions of proven efficacy (agree, uncertain, **disagree**)	352	48.3	43.0–53.6
(3) Genetic tests for breast cancer or colorectal cancer should be performed only if economical evaluations show cost effectiveness ratios favorable compared to alternative health interventions (**agree**, uncertain, disagree)	354	46.3	41.0–51.7
(4) Authoritative and evidence-based guidelines are needed for the appropriate use of genetic tests for breast cancer and colorectal cancer (**agree**, uncertain, disagree)	355	92.4	89.1–94.9
(5) Genetic tests for breast and colorectal cancer should be performed without genetic counseling informing patients of the benefits and risks of the tests (agree, uncertain, **disagree**)	355	76.9	72.2–81.2
(6) Genetic tests for breast and colorectal cancer can contribute efficaciously to health promotion and cancer prevention only if included in wider strategies taking into account the other available health interventions (**agree**, uncertain, disagree)	354	83.1	78.7–86.8
(7) The implementation of genetic tests for breast and colorectal cancer, being a medical matter, should not take into account ethical, legal and social implications (agree, uncertain, **disagree**)	356	75.3	70.4–80.0

Correct answers bolded.

**Table 5:** Self-estimated level of residents' knowledge and training needs on genetic tests for breast and colorectal cancer (*n* = 364).

		*n*	%
How would you rate your level of knowledge on the appropriate use of genetics tests for cancer in clinical practice?	Poor	178	50.6
	Fair	143	40.6
	Good	29	8.2
	Excellent	2	0.6

		Yes	No

How important do you think it is to increase your knowledge about the use of genetics tests for cancer in clinical practice?		309 (87.8%)	43 (12.2%)
Do you find yourself qualified enough to prescribe genetic tests for cancer?		54 (15.4%)	296 (84.6%)
Should more time be dedicated to learning on genetic test during the medical studies?		289 (82.3%)	62 (17.7%)
Should more time be dedicated to learning on genetic test during the medical specialization?		263 (74.7%)	89 (25.3%)
Is there a need for specific postgraduate course on use of genetic testing for cancer?		229 (65.4%)	121 (34.6%)
