# Supplementary material for: Survey on Knowledge, Attitudes, and Training Needs of Italian Residents on Genetic Tests for Hereditary Breast and Colorectal Cancer
Source: Biomed Res Int. 2014 Jun 23;2014:418416. doi: 10.1155/2014/418416 (PMC4094882; doi:10.1155/2014/418416)
Supplement: Supplementary file 1 — Supplementary Table S1 contains a list of medical specializations in Italy according to each particular area. The specializations are placed in three different groups referring to “specializations in area of medicine”, “specializations in area of medicine” and “other specializations”. [file 418416.f1.docx]

| Supplementary Table S1 - List of medical specializations in Italy according to each particular area | |
| --- | --- |
| Specializations in area of medicine | |
| Clinical Allergolgy and Immunology | Dermato-venerology |
| Hematology | Endocrinology |
| Gastroenterology | Geriatrics |
| Cardiology | Pulmonology |
| Infectious disease | Sport medicine |
| Urgent Care Medicine | Internal medicine |
| Thermal Medicine | Tropical medicine |
| Nephrology | Neuropathophysiology |
| Neurology | Pediatrics neuropsychiatry |
| Oncology | Pediatrics |
| Psychiatry | Rheumatology |
| Specializations in field of surgery | |
| Cardiac surgery | Digestive surgery |
| General surgery | Maxillofacial surgery |
| Pediatric surgery | Plastic and reconstructive surgery |
| Thoracic surgery | Vascular surgery |
| Obstetrics and gynecology | Neurosurgery |
| Ophthalmology | Orthopedic and trauma surgery |
| Otorhinolaryngology | Urology |
| Other specializations | |
| Anatomy and histology | Anesthesia and intensive care |
| Audiology and phoniatrics | Pharmacology |
| Genetics in medicine | Hygiene and preventive medicine |
| Occupational Medicine | Physical medicine and rehabilitation |
| Forensic medicine science and pathology | Nuclear medicine |
| Microbiology and virology | Clinical pathology |
| Radio diagnostics | Toxicology |
| Food Science | Radiotherapy |
